# Urinary tract infection in patients with diabetes mellitus and the role of parental genetics in the emergence of the disease

**DOI:** 10.25122/jml-2021-0331

**Published:** 2022-08

**Authors:** Heyam Emad Al Qurabiy, Ihab Majeed Abbas, Aboo-Thar Ali Hammadi, Farah Kadhim Mohsen, Rasha Ibrahim Salman, Saja Hussain Dilfy

**Affiliations:** 1 Department of Medical Laboratory Techniques, Kut University College, Al-Kut, Iraq; 2 Department of Biology, College of Education for Pure Science, Wasit University, Al-Kut, Iraq

**Keywords:** diabetic mellitus, UTI, parental genetic, family history, bacterial isolates

## Abstract

This study aimed to assess the role of paternal genetics in the development of diabetic mellitus (DM) and determine the impact of DM on the urinary system by investigating the percentage of patients with urinary tract infection (UTI). The study included 100 people with DM; their ages ranged from 5 to 83 years. The DM and blood sugar levels were diagnosed clinically and at a laboratory in Al-Zahra Teaching Hospital and the outpatient clinics. The age, gender, and causes of DM and the family history of diabetes were reported. Isolation and identification of bacterial species were made depending on culture media and biochemical tests. The average age of patients was 47.7±5.5, and most of them were female (67%). The incidence of DM increased with age, and the main cause of DM was likely to be a genetic predisposition (family history), where 32% of patients appeared to have a positive family history and the presence of DM in both parents or only the mother had a significant role in increasing the genetic predisposition of developing DM. Among the non-genetic causes of DM, the most common was exposure to sudden psychological or nervous shock (41%). Obesity also had an important role in the development of diabetes, and also pregnancy and smoking. Moreover, 66% of patients with type 2 DM and all with type 1 DM suffered from UTIs. The main causative agents were *E. coli* (60%) and *Proteus spp*. (13%). The majority of patients suffering from UTIs (73%) were females. In conclusion, type 2 DM is the most common, especially in females, and increases with age. The main cause of DM was family genetic predisposition and sudden shocks. The current study also showed that most diabetic patients suffered from UTIs, especially females, and the main causes of UTI inflammation are *E. coli* isolates.

## INTRODUCTION

Diabetes mellitus is a metabolic illness defined by unusually high blood sugar levels caused by a lack of the hormone insulin, impaired tissue sensitivity to insulin, or both [1]. Diabetes causes serious complications or even death at a young age. However, a diabetic patient can control the disease and reduce the risk of complications by taking certain steps [2]. These steps include reducing weight and increasing movement. The World Health Organization divided diabetes into three main types: type 1 diabetes, type 2 diabetes, and gestational diabetes. Each type has its causes and is distributed worldwide [3]. There are rare causes of diabetes that cannot be classified as type 1 or type 2 diabetes in pregnant women and attempts to classify diabetes cause much controversy among specialists [4]. In type 1 diabetes, immune cells in the human body attack the beta cells of the pancreas, which produce insulin, and the reason for this is still unknown, but the reason is sometimes attributed to the infection of children with pathological infections at a young age, adding to genetic may play a role in the occurrence of disease [5, 6]. After a meal, starches are broken down during metabolism into a sugar called glucose, delivered by the blood to all body cells for consumption and energy generation. People with diabetes have trouble converting food into energy for metabolism. Insulin is required by most body cells for glucose from the blood to reach the cells [7, 8]. Consumption of foods high in sugar and starches when the liver and pancreas cannot produce enough insulin to enter sugar into cells, causing a portion of the sugar to remain in the blood, represents type 2 diabetes [9]. It causes an overabundance in the blood while the cells remain hungry for energy.

Hyperglycemia develops over time, causing serious nerve and blood vessel damage that can lead to consequences like heart disease, stroke, kidney disease, blindness, diabetic neuropathy, gum infections, diabetic foot, and even amputation [10, 11]. Furthermore, diabetes mellitus is linked to glycosuria, poor immunity, and bladder dysfunction, all of which are considered risk factors for UTIs [12, 13]. Gestational diabetes is a disorder in which blood sugar levels rise during pregnancy and is classified as type 2 diabetes affecting up to 10% of pregnant women in the United States [14]. Gestational diabetes affects pregnant women who have never been diagnosed with diabetes. There are two types of gestational diabetes. Women in group A1 can control their symptoms with diet and exercise, but women in category A2 require insulin or other drugs [15, 16].

Earlier research indicated that heredity has a role in the development of diabetes and a family history of the condition is a classic risk factor linked to the disease [17]. However, whether the mother's or father's family history is the most essential or risky factor in the development of diabetes is still a point of contention [18].

Many previous studies focused on the role of the immune response to diabetes and its relationship to circulatory disorders. According to our knowledge, there is no study in Iraq to determine the role of family history as a cause of diabetic mellitus and the incidence and causes of urinary tract infection in diabetic patients.

## Material and Methods

### Patients and study design

The current cross-sectional study was conducted among patients with DM admitted to Al-Zahra Teaching Hospital and outpatient clinics from January to March 2021. We collected blood and urine samples and other relevant information from 100 diabetic patients diagnosed at Biomedical Bacteriologic and Hematology Labs. Each patient provided the following information:

1. Name and gender;
2. Age;
3. Diabetes interval;
4. Diabetes family history;
5. Urinary tract infection;
6. Treatment;
7. Pregnancy.

### Sample collection and preparation

1–2 cc of blood sample was collected from patients, placed in a plain tube, and left for 5–10 minutes until clotting. Following this, it was placed in the centrifuge and set to revolve at 3000 revolutions per minute for 5 minutes to separate the serum from the blood sample. Urine samples were also collected in sterile urine caps labeled with the patient's information.

### Methods for testing blood sugar

The blood sugar level was measured using two types of tests: fasting blood sugar, the test after 8–12 hours of eating, and the random blood sugar test. Blood sugar was measured after an overnight fast in a fasting blood sugar test (not eating). A fasting blood sugar level of 99 mg/dL or less was considered normal, while 100 to 125 mg/dL was considered prediabetes, and 126 mg/dL or more was considered diabetes. A random glucose test is a quick test that can be performed in a lab or clinic at any time. There is no need for the person to fast beforehand. A sterile needle was used to take a small blood sample from the finger. A blood sugar level of 200 mg/dL or higher indicates the possibility of diabetes. However, we repeated this diagnosis on a different day to ensure a more accurate diagnosis. Some patients used the A1C test to determine their average blood sugar level over the previous two or three months. Normal A1C was less than 5.7 percent, prediabetes was between 5.7 and 6.4 percent, and diabetes was 6.5 percent or higher.

### Urinary diabetes test

We took a urine sample and used urinalysis reagent strips. We put the tape in the urine for 20 seconds, extracted it, and left it for 60 seconds. Following this, we compared the colors on the box.

### Diagnosis of urinary tract infection

Patients with urinary tract infections were identified at Al-Zahra Teaching Hospital labs and external laboratories, with most patients suffering from bacterial infections. Others suffered from harm caused by stones or sand in the kidneys and bladder defects. All patients had antibiotics, and a substantial percentage developed recurring UTIs. Bacterial species were isolated and diagnosed using culture medium and biochemical tests (Table 1).

**Table 1:** Culture media used for isolation of bacterial species.

Culture media	Manufacturer
**Blood agar**	Sterelline (UK)
**MacConkey agar**	Accumix (India)
**Mannitol salt agar**	Sterelline (UK)
**Eosin methylene blue**	Accumix (India)
**Hektoen enteric agar**	Accumix (India)

### Statistical Analysis

The data were analyzed using Excel 2010 and the Statistical Package for Social Sciences (SPSS 19) software, with results considered statistically significant if the P-value was less than 0.05.

## Results

**Figure 1:**
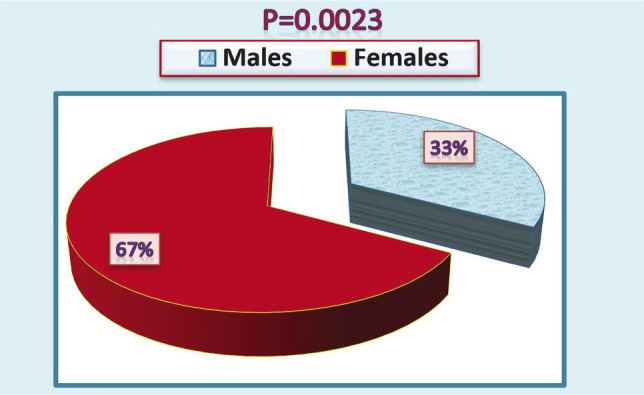
Distribution of diabetic patients according to gender.

**Figure 2:**
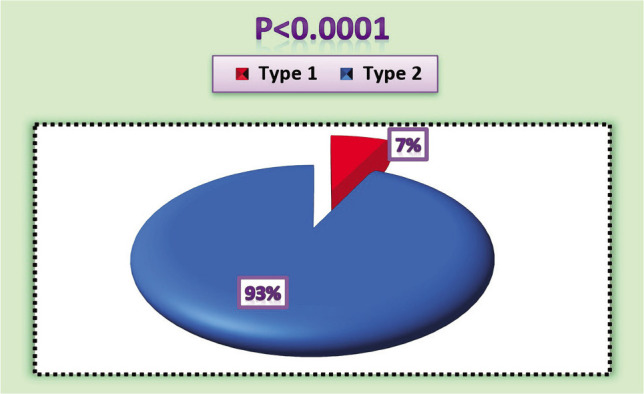
The type of diabetes mellitus among patients.

The current study collected blood samples from 100 diabetic patients diagnosed clinically and in the laboratory. Their ages ranged from 5 to 83 years, with an average age of 47.7±5.5 years, as shown in Table 2. Most of the patients were females (67%), and 33% were males (Figure 1). We divided the ages of patients into four age groups, as in Table 2. The majority of males (59%) with diabetes were within the age group ≤60, while most females (69%) were in the age group of 30–45 years. Type 2 diabetes was the most common (93%) (93 patients) compared to the first type. However, the symptoms may be less clear for type 1 diabetes, so the patient may be diagnosed several years after the onset of symptoms, that is, when the patient reaches the age of 20 years, *i.e*., after complications occur. Studies confirm the emergence of this pattern during childhood, as seen in 7% of patients in our study (p<0.0001), as in Figure 2.

**Table 2:** Distribution of diabetic patients according to age and gender.

Cases Number	Age Range	Mean±SD	Female	Male	P-value
			N (%)	N (%)	
**Total N=100**	5–83	47.7±5.5	67 (67)	33 (33)	0.0028*
**10**	<15	5±2.13	5 (50)	5 (50)	1
**16**	15–30	3.7±18.5	9 (56)	7 (44)	0.123
**16**	30–45	38.3±4.1	11 (69)	5 (31)	0.0023*
**26**	45–60	51.5±5	13 (50)	13 (50)	1
**32**	≥60	69±8.3	13 (41)	19 (59)	0.046*

* – significant (p<0.05); SD – Standard Deviation; N – Number.

**Figure 3:**
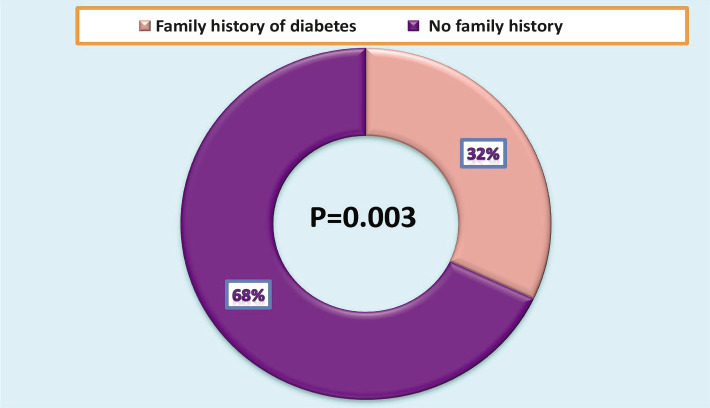
Family history of diabetes mellitus.

**Figure 4:**
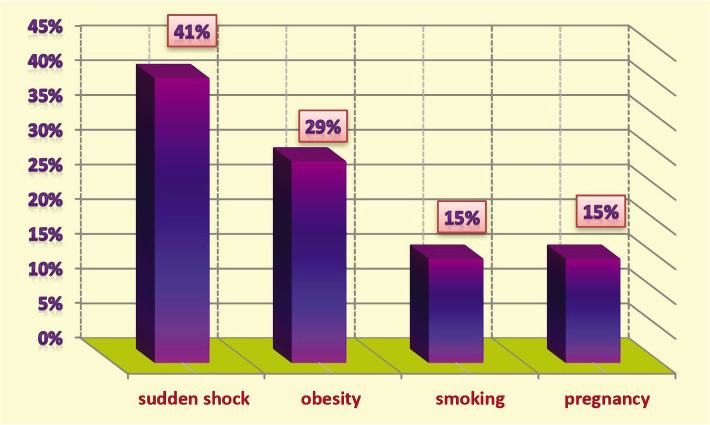
Non-genetic causes of diabetic mellitus.

The main cause of diabetes was a genetic predisposition (family history) (Figure 3), with 32% of patients having a positive family history of diabetes. Furthermore, 68% of DM patients had DM due to various other factors, as shown in Figure 4. Among the non-genetic causes of diabetes, the most common was a sudden nervous or psychological shock (41%), obesity (29%), while smoking and pregnancy had the same effect (15% for each of one) (Figure 4).

43% of patients with type 1 diabetes had a positive family history (29% from both parents and 14% from mother only) (Table 3). Furthermore, 31% of type 2 diabetes patients had a positive family history. Among these patients, 16% had a positive family history for the mother and father, while 11% with a positive family history only for the mother, while 4% of type 2 diabetes patients had a family history for the father only.

**Table 3:** Distribution of diabetes mellitus according to inheritance pattern.

Diabetic types	Positive family history	Mother & father	Mother	Father	P-value
	N (%)	N (%)	N (%)	N (%)	
**Type 1 (n=7)**	3 (43)	2 (29)	1 (14)	0 (0)	0.003*
**Type 2 (n=93)**	29 (31)	15 (16)	10 (11)	4 (4)	0.0411*

* – significant analysis (p<0.05).

**Figure 5:**
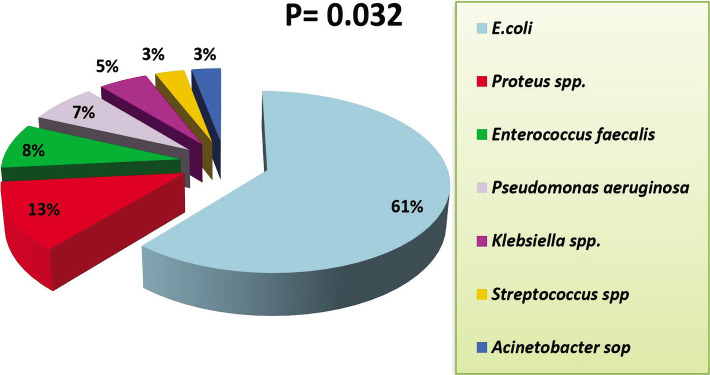
Bacterial isolates diagnostic.

The results of the current study showed that all patients with type 1 DM suffered from UTI, and 66% of patients with type 2 diabetes suffer from UTIs, as shown in Table 4. Current studies also showed that most diabetic females suffer from urinary tract infections with a percentage of 73%, compared to males, at a rate of 30%. According to culture and biochemical tests, *E. coli* (61%) was the most common cause of UTI, followed by *Proteus spp*., *Enterococcus faecalis, Pseudomonas aeruginosa*, and *Klebsiella* (13%, 8%, 7%, and 5%, respectively), with fewer other isolates (Figure 5).

**Table 4:** UTI among diabetic patients.

Diabetic types	Total number (%)	UTI	Non-UTI	P-value
		N (%)	N (%)	
**Type 1**	7 (7)	4 (100)	0 (0)	<0.0001*
**Type 2**	93 (93)	61 (66)	32 (34)	0.0015*
**Patients Gender**	**Total number (%)**	**N (%)**	**N (%)**	**P-value**
**Female**	67 (67)	49 (73)	18 (27)	0.0041*
**Males**	33 (33)	10 (30)	23 (70)	0.0050*

* – significant value (p<0.05).

## Discussion

DM appears at various ages, from 5 to 83 years. The exact cause of diabetes is unknown, but some studies have shown that diabetes (type I) in children is the result of the immune system destroying the insulin-producing cells (beta cells) in the pancreas by mistake [19, 20]. Once the pancreatic cells are destroyed, the baby produces less or no insulin, and as a result, sugar builds up in the bloodstream, which can cause life-threatening complications [7–20]. The majority of diabetic patients in the current study were adults, indicating that type 2 diabetes was the most common. This finding is consistent with previous research, which found that adult-type or type 2 diabetes accounts for 85–90 percent of cases and is caused by a defect in the body's cells' ability to respond to insulin completely. This decreases their ability to exploit glucose from the blood, medically known as insulin resistance.

In other words, the cells need a greater amount of insulin to respond as required [21–23]. Some studies showed that type 2 diabetes also occurs due to the body's inability to use insulin, and most of the time as a result of being overweight and physical inactivity, with symptoms similar to those of type 1. However, because the symptoms may be less obvious in many cases, the patient may be diagnosed several years after the onset of symptoms, that is, when the patient reaches the age of 20, *i.e*., after complications, as shown in the current study. Other studies confirm the emergence of this pattern in childhood [24–26].

The incidence of diabetes was higher in females, which agrees with previous studies that mentioned that the risk of diabetes increases in women after 40 years. However, this study does not agree with studies that recorded an increased prevalence in males and justified that by increasing fats in the abdominal area in men, causing an increase in the resistance of cells to insulin [27–29]. On the other hand, one study showed that women are more likely to develop diabetes than males due to greater exposure to psychological problems and pressures than men and their strong reaction when exposed to some shocks [30, 31]. Another American study stated that males are more affected by type 2 than women, but women have more negative consequences due to the changes that women experience during their lifetime, such as pregnancy, breastfeeding, menopause, and hormonal disorders [32, 33]. According to recent studies on diabetes in women, polycystic ovarian syndrome tends to increase the risk for type 2 diabetes, characterized by an increase in the size of the ovaries and the inability to release eggs properly [34–36].

The results of the current study show that genetic predisposition plays a major role in the emergence of diabetes, and this result is consistent with a study conducted by Fuchsberger and his colleagues in 2016, which stated that the chance of developing type 1 or type 2 diabetes increases if one of the parents or grandparents have diabetes [37, 38]. The genetic predisposition means that there is a mutation in the genetic code responsible for the production of insulin, or there may be a genetic mutation in the genes encoding the insulin receptors in the body tissues of the affected person [39, 40]. Previous research also mentioned that environmental factors increase the stimulation of genetic factors. Studies also show that genetics plays a partial role in the patient's suffering with the first and second types [41, 42]. Type 1 diabetes is most often caused by an infection (mostly viral) or other forms of stimulation on a small scale, such as psychological stress and exposure to surrounding environmental factors, such as certain chemicals or medications.

Furthermore, several hereditary factors influence an individual's reaction to these stimuli [43, 44]. Harder *et al*. observed that among women with gestational diabetes, a family history of type 2 diabetes was more prevalent in the maternal and grand-maternal lines than in the paternal and grand-paternal lines [45]. However, that study was constrained since it did not include a control group with normal glucose tolerance, the number of study participants was small, and the diagnostic criteria employed were out of date [46]. Genetic variables, intrauterine nutritional status, and shared environmental factors are all possible pathways that might explain the relevance of maternal history. Some nuclear genes are inherited preferentially from the mother, while mitochondrial DNA mutations or deletions are inherited completely from the mother [47, 48].

The current study also showed that psychological and neurological disorders play a role in diabetes due to the effect of neural signals on insulin secretion. This type of diabetes also appeared in some pregnant women, often in the second and third stages of pregnancy [49, 50]. Previous studies showed that half of the women exposed to gestational diabetes continue with the second type after pregnancy [51]. In the current study, body weight was one of the leading causes of diabetes, preceded by genetic predisposition and exposure to severe shock. The main factors in the current study were genetic predisposition and exposure to sudden shock, with body weight being one of the most important causes of diabetes [52, 53]. This is consistent with previous studies that confirmed that body weight (obesity) is one of the most important factors affecting the incidence of diabetes due to the accumulation of fatty tissue in the body, which increases as the cells' resistance to insulin is greater [54]. A study in 2015 showed that if the fatty tissue is in the upper part more than in other areas of the body, especially around the abdomen, there is a greater risk of developing diabetes [55].

This study also showed that smoking plays a role in the occurrence of diabetes, and this is consistent with recent scientific studies that revealed that smoking increases the level of glucose in the blood, weakening the control of diabetic patients. The increase of nicotine in the body and its impact on the efficiency of the pancreas performance responsible for the secretion and production of hormone insulin results in a lack of insulin secretion, leading to a high glucose level in the blood [56–58].

Diabetic patients are at increased risk of infection in general and, in particular, UTIs. The predisposition to UTIs among diabetic patients results from multiple factors, the length of the disease and its severity [59, 60], the high content of glucose in the urine, and the defect in the patient's immune factors. Furthermore, the increase in blood sugar causes disturbance in the function of neutrophils by increasing the level of calcium inside the cell and the effect on actin (a protein involved in many types of eukaryotic cells and forms filaments that form the main component of support for the cytoskeleton) then on diapedesis and phagocytosis over time. Finally, diabetic patients may develop urinary bladder disease, nephropathy, or renal papillary necrosis and complications that predispose them to urinary tract infection [61, 62]. In the present study, the main causes of UTI were E.coli and *Proteus spp*., *Enterococcus faecalis, Pseudomonas aeruginosa, Klebsiella spp*., *Streptococcus spp*., and *Acinetobacter spp*., which appeared in low rates. One study in Sudan showed that the predominant isolates were *E. coli* (56.4%) and *K. pneumoniae* (23%) in patients with UTIs and found a high incidence of UTI and asymptomatic bacteriuria among diabetic patients compared with non-diabetic patients [63].

Moreover, some studies showed no significant difference in the frequency of isolated microorganisms between diabetic and non-diabetic patients with UTIs. In this study, the most frequently isolated microorganism was *E. coli*, with a rate of 60%, which agrees with previous studies [64, 65]. The higher incidence of *E. coli* could be related to the fact that they are commensals of the bowels, and there could be fecal contaminations due to poor hygiene and their unique structure, which promote colonization of the host epithelial cells within the urinary tract and prevent bacteria from urinary washing [66].

Patient age, gender, duration, and type of DM were associated with the prevalence of UTIs in the current study. This finding differed from findings reported for diabetic patients in Saudi Arabia [67–70] but supports other studies showing that older age, duration of DM, and type of diabetic mellitus are risk factors for UTI among diabetic patients [71–75].

## Conclusion

Type 2 diabetes is more common, especially in females, and increases with age. The current research also showed that the main causes of diabetes are a genetic predisposition to DM, sudden psychological or nervous shock, obesity, smoking, and pregnancy. The family history of both parents or only the mother plays an important role in increasing the offspring's predisposition to diabetes. According to culture and biochemical tests, the main causes of UTI are *E. coli* and *Proteus spp*. Moreover, all patients with type 1 diabetes suffered from UTIs, while 66% of patients with type 2 diabetes had UTIs, and the highest percentage appeared in females (73%).
